# Association Between Visceral Adiposity and the Prediction of Hepatic Steatosis and Fibrosis in Patients with Metabolic Dysfunction-Associated Steatotic Liver Disease (MASLD)

**DOI:** 10.3390/jcm14103405

**Published:** 2025-05-13

**Authors:** Renata Bende, Darius Heredea, Iulia Rațiu, Ioan Sporea, Mirela Dănilă, Roxana Șirli, Alina Popescu, Felix Bende

**Affiliations:** 1 Department of Gastroenterology and Hepatology, “Victor Babes” University of Medicine and Pharmacy Timisoara, Eftimie Murgu Square 2, 300041 Timisoara, Romania; bende.renata@umft.ro (R.B.); ratiu.iulia@umft.ro (I.R.); isporea@umft.ro (I.S.); danila.mirela@umft.ro (M.D.); sirli.roxana@umft.ro (R.Ș.); popescu.alina@umft.ro (A.P.); bende.felix@umft.ro (F.B.); 2 Advanced Regional Research Center in Gastroenterology and Hepatology, “Victor Babes” University of Medicine and Pharmacy Timisoara, 300041 Timisoara, Romania

**Keywords:** MASLD, fibrosis severity, non-invasive markers

## Abstract

**Background/Objectives**: Metabolic dysfunction-associated steatotic liver disease (MASLD) is a major cause of chronic liver disease and is closely linked to obesity and metabolic syndrome, necessitating efficient, non-invasive diagnostic tools. **Methods**: This monocentric cross-sectional study included 178 patients (69.1% with MASLD, 30.9% normal subjects; 55% males; mean age 52.79 ± 12.56 years) who underwent anthropometric and biochemical assessments to determine the visceral adiposity index (VAI), triglyceride–glucose index (TyG), and lipid accumulation product (LAP), along with abdominal ultrasound and vibration-controlled transient elastography (VCTE) with controlled attenuation parameter (CAP). **Results**: Patients were categorized based on steatosis severity: S0–S1 (n = 64) and S2–S3 (n = 114). The TyG, VAI, and LAP values were significantly higher in S2–S3 cases (*p* < 0.0001) and showed moderate-to-strong correlations with both steatosis and fibrosis. Predictive models yielded AUROCs of 0.80 (TyG), 0.83 (VAI), and 0.79 (LAP) for diagnosing S2–S3 steatosis. The NAFLD fibrosis score (NFS) and FIB-4 classified fibrosis severity, but 36.8% of cases remained unclassified. Applying the TyG and VAI thresholds reduced this rate to 26.3%. **Conclusions**: These findings support the TyG, VAI, and LAP as valuable non-invasive biomarkers for MASLD assessment, enhancing the classification accuracy when conventional fibrosis scores are inconclusive.

## 1. Introduction

Metabolic dysfunction-associated steatotic liver disease (MASLD) is the most recent term used to describe steatotic liver disease linked to metabolic syndrome [1]. MASLD is estimated to affect 30% of the adult population worldwide, with its prevalence increasing from 22% to 37% from 1991 to 2019 [2,3]. The rising prevalence of MASLD mirrors the growing rates of obesity and obesity-related conditions. As the leading cause of chronic liver disease, MASLD significantly contributes to liver-related morbidity and mortality. Tackling the public health issue of obesity and its related conditions, including MASLD, necessitates coordinated efforts from general practitioners and specialists from various fields. Establishing a straightforward and accessible assessment and referral pathway that utilizes non-invasive tests is crucial to accurately and promptly identify patients with MASLD who need to be referred to a specialist [4].

As obesity and related metabolic disorders burgeon worldwide, understanding the nuanced relationship between these conditions and hepatic steatosis becomes imperative. In the labyrinth of metabolic intricacies, researchers continue to unveil the profound interplay between various non-invasive markers and the presence of complications associated with metabolic syndromes, such as MASLD.

A range of non-invasive markers, such as biological markers (glucose levels, cholesterol, triglycerides, gamma-GT, cytokine 18), BMI, abdominal circumference, or various combinations thereof, have been evaluated for their interdependent relationship with the presence and severity of hepatic steatosis [4,5,6]. Studies underline that the diagnostic criteria used to define metabolic syndrome significantly influence the observed risk of hepatic steatosis and fibrosis. For instance, a retrospective analysis of over 2000 patients demonstrated that, irrespective of the definition applied, the presence of metabolic syndrome increased the probability of hepatosteatosis by up to 36% and hepatofibrosis by up to 47%, with central obesity and hypertriglyceridemia emerging as the strongest individual predictors [7]. These findings further support the importance of refining diagnostic strategies and non-invasive tools to better capture liver-related complications in metabolically at-risk populations.

In recent years, the visceral adiposity index (VAI), triglyceride–glucose index (TyG), and lipid accumulation product (LAP) have emerged as novel indices, offering insights into metabolic health beyond traditional measures. The LAP, a marker integrating waist circumference and triglyceride levels, offers a robust tool for assessing lipid over-accumulation and associated metabolic risks.

The VAI, incorporating waist circumference, triglycerides, high-density lipoprotein cholesterol, and fasting glucose, offers a nuanced portrayal of visceral adiposity and metabolic dysfunction. The TyG, a product of fasting triglycerides and glucose levels, reflects insulin resistance and has garnered attention for its predictive value in metabolic disorders [8,9,10,11].

Recent evidence highlights the crucial role of gut microbiota alterations in the development of metabolic syndrome and MASLD. Dysbiosis promotes systemic inflammation, insulin resistance, and metabolic disturbances, while changes in the gut microbiota composition correlate with metabolic biomarker alterations [12,13]. Furthermore, interactions between the gut microbiota, host genetics, and epigenetic modifications contribute to disease progression and the development of MASLD-related hepatocellular carcinoma, emphasizing the importance of integrative approaches [14].

Given the increasing understanding of the multifactorial mechanisms underlying MASLD, the identification of reliable, simple, and non-invasive clinical predictors becomes essential to facilitate the early diagnosis, risk stratification, and management of affected individuals.

The aim of this study is to evaluate the performance of the visceral adiposity index (VAI), lipid accumulation product (LAP), and triglyceride–glucose index (TyG) in accurately identifying patients with hepatic steatosis and assessing the risk of hepatic fibrosis in individuals with metabolic syndrome.

## 2. Materials and Methods

### 2.1. Study Population

**Figure 1:**
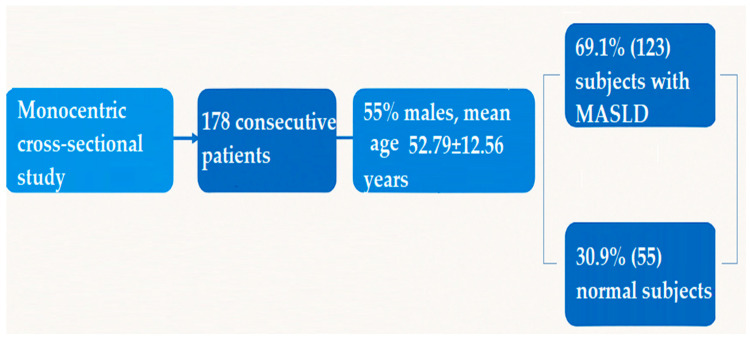
Flowchart of the inclusion criteria.

A monocentric cross-sectional study was performed during a 2-year interval in a tertiary Department of Gastroenterology and Hepatology. Patients who met this study’s inclusion criteria were identified, and a total of 178 consecutive patients (Figure 1) were included.

This study’s inclusion criteria were age over 18 years, agree to participate in the study, and signed informed consent.

For the MASLD group (n = 122), participants were included if they had evidence of hepatic steatosis—confirmed by abdominal ultrasound and/or controlled attenuation parameter (CAP) > 248 dB/m—in association with at least one criterion of metabolic dysfunction, as defined by the recent MASLD consensus. These criteria included the following:

- Body mass index (BMI) ≥ 25 kg/m^2^;
- Type 2 diabetes mellitus;
- At least two of the following metabolic risk factors:
  - Waist circumference ≥ 102 cm in men or ≥88 cm in women;
  - Blood pressure ≥ 130/85 mmHg or current antihypertensive treatment;
  - Triglycerides ≥ 150 mg/dL or specific treatment for hypertriglyceridemia;
  - HDL-cholesterol < 40 mg/dL in men or <50 mg/dL in women;
  - Fasting plasma glucose between 100 and 125 mg/dL or HbA1c ≥ 5.7%.

For the control group (n = 56), participants were included only if they met all of the following criteria:

- Absence of hepatic steatosis, confirmed both by abdominal ultrasound and CAP < 248 dB/m;
- BMI < 25 kg/m^2^;
- No diagnosis of type 2 diabetes mellitus;
- No more than one metabolic risk factor from the list detailed above.

Participants were excluded from this study if they met any of the following criteria: absence of informed consent, age under 18 years, failure to meet any metabolic syndrome criteria, consumption of alcohol in toxic doses (defined as >210 g/week for men and >140 g/week for women), presence of a clearly defined chronic liver disease (such as hepatitis B or C, autoimmune hepatitis, primary biliary cholangitis, or primary sclerosing cholangitis), presence of ascites or biliary obstruction, aminotransferase levels exceeding five times the upper normal limit, history of cancer, detectable focal liver lesions, or heart failure leading to hepatic congestion. All the patients provided written informed consent before study entry. This study was conducted in accordance with the guidelines of the Declaration of Helsinki and approved by the Ethics Committee of the County Hospital “Pius Brinzeu” from Timisoara with approval number 181/5 December 2022.

### 2.2. Clinical and Laboratory Examinations

Demographic data were collected from all the included subjects. All the included subjects underwent the following: anthropometric measurements, biological tests (complete blood count, fasting blood glucose levels, cholesterol, HDL-cholesterol, LDL-cholesterol, triglycerides, gamma-GT, serum albumin levels, aspartate transferase (AST) levels, alanine transaminase (ALT) levels), B-mode abdominal ultrasound, vibration-controlled transient elastography (VCTE) with controlled attenuation parameter (CAP) (FibroScan, EchoSens, Paris, France), VAI, TyG index, LAP, nonalcoholic fatty liver disease fibrosis score (NFS), and FIB-4 index determinations.

Anthropometric measurements included body weight (measured using a calibrated digital scale with participants wearing light clothing and no shoes), height (measured using a standard measuring tape), and waist circumference (measured at the midpoint between the lower margin of the last palpable rib and the top of the iliac crest, using a non-elastic tape, with the participant standing and breathing out gently).

Laboratory analyses were performed in the hospital’s clinical laboratory, where standardized protocols and certified equipment were used. The parameters evaluated from fasting venous blood samples are summarized in Table 1.

The nonalcoholic fatty liver disease fibrosis score (NFS) was determined using the established equation: −1.675 + 0.037 × age (in years) + 0.094 × BMI (kg/m^2^) + 1.13 × (presence of impaired fasting glucose or diabetes, coded as yes = 1, no = 0) + 0.99 × AST/ALT ratio − 0.013 × platelet count (×10⁹/L) − 0.66 × serum albumin (g/dL). For interpretation, values above 0.676 were considered indicative of significant fibrosis (rule-in), while those below −1.455 were used to rule out advanced fibrosis [15].

The FIB-4 index was calculated using the following formula: FIB-4 = Age (years) × AST (U/L)/[PLT (109/L) × ALT1/2 (U/L)]. The following cut-off values were used: rule-in cut-off value FIB-4 > 2.67, and rule-out cut-off value FIB-4 ≤ 1.3 [16].

The values for VAI, TyG, and LAP were computed using the collected data. The TyG index, which is the natural logarithm of the product of fasting triglycerides (TG) and body mass index (BMI), was calculated using the formula: ln[BBG (mg/dL) × TG (mg/dL)/2]. The TyG index has been proposed as an alternative marker for insulin resistance (IR) due to its correlation with lipotoxicity and glucotoxicity. Reported normal cutoff values for the TyG index in the medical literature range widely between 4 and 8, attributable to the placement of 2 within the TyG index formula.

The lipid accumulation product (LAP), an established indicator of visceral fat accumulation, was calculated based on waist circumference (WC) and fasting triglyceride (TG) levels. The calculation formulas are as follows: for men, LAP = (WC in cm − 65) × TG (mmol/L), and for women, LAP = (WC in cm − 58) × TG (mmol/L). Reference cut-off values for the LAP typically range from 25.16 to 31.59 cm × mmol/L in women and from 20.10 to 63.89 cm × mmol/L in men. The LAP is widely applied as a surrogate marker for both abdominal obesity and metabolic syndrome.

The visceral adiposity index (VAI) is a mathematical model that incorporates both anthropometric measurements—such as body mass index (BMI) and waist circumference (WC)—and biochemical markers including triglycerides (TG) and high-density lipoprotein cholesterol (HDL). It is used to estimate visceral fat function and its relationship with insulin resistance. The VAI was calculated using gender-specific formulas: for men, VAI = (WC in cm)/[39.68 + (1.88 × BMI)] × (TG/1.03) × (1.31/HDL); for women, VAI = (WC in cm)/[36.58 + (1.89 × BMI)] × (TG/0.81) × (1.52/HDL) [17].

### 2.3. Vibration-Controlled Transient Elastography and Controlled Attenuation Parameter

All participants underwent vibration-controlled transient elastography (VCTE) and controlled attenuation parameter (CAP) measurements using the FibroScan^®^ Compact 530 system (EchoSens, Paris, France), with either the standard M probe (3.5 MHz) or the XL probe (2.5 MHz), depending on the automated probe selection tool’s recommendation. The result was considered reliable when the interquartile range to median value ratio (IQR/M) was below 30%, calculated from the median of 10 valid readings. Liver stiffness was expressed in kilopascals (kPa), within a range of 2.5–75 kPa, and steatosis levels were measured in decibels per meter (dB/m), ranging from 100 to 400 dB/m [18].

Participants were asked to fast for at least four hours prior to the procedure. Assessments were performed with the subject lying in a supine position, right arm fully extended above the head, after resting for a minimum of 10 min. The probe was applied intercostally and aligned parallel to the rib spaces. Individuals showing TE values under 6 kPa and CAP values below 248 dB/m were categorized as having normal liver findings. If these thresholds were exceeded and the individuals also met the previously defined criteria for metabolic syndrome, they were classified as having MASLD [19,20].

This study employed the cut-off values recommended by Eddowes et al. (2019) for NAFLD patients, using liver biopsy as the gold standard: F2 ≥ 8.2 kPa, F3 ≥ 9.7 kPa, and F4 ≥ 13.6 kPa [21], with 8.2 kPa considered indicative of significant fibrosis (F ≥ 2). For steatosis, we used the cut-off values from Petroff et al. (2021): S1 ≥ 294 dB/m, S2 ≥ 310 dB/m, and S3 ≥ 331 dB/m, with 310 dB/m indicative of significant steatosis (S2–S3) [22].

### 2.4. Statistical Analysis

Statistical analyses were performed using MedCalc Version 19.4 (MedCalc Software Corp., Brunswick, ME, USA) and Microsoft Office Excel 2019 (Microsoft for Windows). Descriptive statistics were applied to summarize demographic, anthropometric, and clinical data. Sample size determination was based on α as the level of significance and Z 1 − α/2 from the standard normal distribution.

A power analysis was conducted using G*Power 3.1 software. Assuming a moderate effect size (Cohen’s d = 0.5), an alpha level of 0.05, and a desired power of 0.80, the minimum required sample size was calculated to be 128 participants.

The Kolmogorov–Smirnov test was used to assess the distribution of numerical variables. Normally distributed continuous variables are presented as means with standard deviations (SDs), while non-normally distributed variables are expressed as medians with interquartile ranges (IQRs). Categorical variables are reported as frequencies and percentages. Group comparisons were performed using Student’s *t*-test for normally distributed variables, the Mann–Whitney U-test for non-normally distributed variables, and Pearson’s χ^2^-test for categorical variables.

Associations between the TyG index, VAI, LAP, and liver steatosis or fibrosis were analyzed using Spearman’s correlation test. A *p*-value < 0.05 was considered statistically significant. Univariate and multivariate regression analyses were conducted to identify predictors of hepatic steatosis. Multivariate models were developed using the Akaike criterion, and predictors were selected using a backward-stepwise algorithm (inclusion *p* < 0.05, exclusion *p* > 0.10). Model accuracy was validated using R-squared values.

The predictive performance of parameters for liver steatosis and fibrosis was evaluated by calculating the areas under the receiver operating characteristic (AUROC) curves. Optimal cut-off values were identified using Bayesian analysis to maximize sensitivity and specificity while minimizing misclassification. Rule-in and rule-out cut-off values were also determined. The positive predictive value (PPV), negative predictive value (NPV), and diagnostic accuracy were calculated, along with 95% confidence intervals (CIs). A *p*-value < 0.05 was considered statistically significant throughout.

## 3. Results

One hundred seventy-eight consecutive subjects (69.1% (123) subjects with MASLD, 30.9% (55) normal subjects), 55% males, mean age 52.79 ± 12.56 years, were included. Subjects were divided into two groups: subjects with no steatosis or mild steatosis S0–S1 (n = 64) and subjects with moderate and severe steatosis S2–S3 (n = 114). The baseline characteristics, demographic data, laboratory parameters, and CAP values of the patients are summarized in Table 2. We also performed an analysis of the steatosis and fibrosis groups according to age and gender, and no significant differences were found between the groups. The analysis is summarized in Table 3.

Upon analyzing the statistical correlations between the non-invasive markers (LAP, TyG, and VAI) measured in the included subjects and both the presence and severity of steatosis and fibrosis, moderate to strong correlations were identified. Notably, the markers demonstrated slightly stronger associations with liver steatosis compared to liver fibrosis (Table 5).

Furthermore, univariate and multivariate statistical analyses were used to examine the relationships between liver steatosis and fibrosis and the following parameters: TyG index, VAI, LAP, age, sex, body mass index (BMI), aspartate aminotransferase (AST), alanine aminotransferase (ALT), low-density lipoprotein cholesterol (LDL-C), high-density lipoprotein cholesterol (HDL-C), fasting blood glucose (FBG), and triglycerides. Univariate analysis showed that the TyG (*p* = 0.002 for steatosis, *p* = 0.0256 for fibrosis), VAI (*p* < 0.001 for steatosis, *p* = 0.011 for fibrosis), and LAP values (*p* = 0.031 for steatosis, *p* = 0.0386 for fibrosis) were significantly associated with both liver steatosis and fibrosis.

In addition, BMI, abdominal circumference, LDL-C, and FBG were also significantly associated with the presence of liver steatosis in the univariate analysis (*p* < 0.05) but not with fibrosis. Using these biomarkers and clinical variables as predictors, a multivariate direct logistic regression analysis was performed to construct two predictive models: one for liver steatosis and one for liver fibrosis. In the final multivariate regression model for predicting liver steatosis, the variables that remained independently associated were the TyG index, VAI, LAP, and BMI. In contrast, the multivariate model for liver fibrosis retained only the TyG index and VAI as independent predictors. The final models, obtained through a stepwise selection approach, are summarized in Table 6.

**Figure 3:**
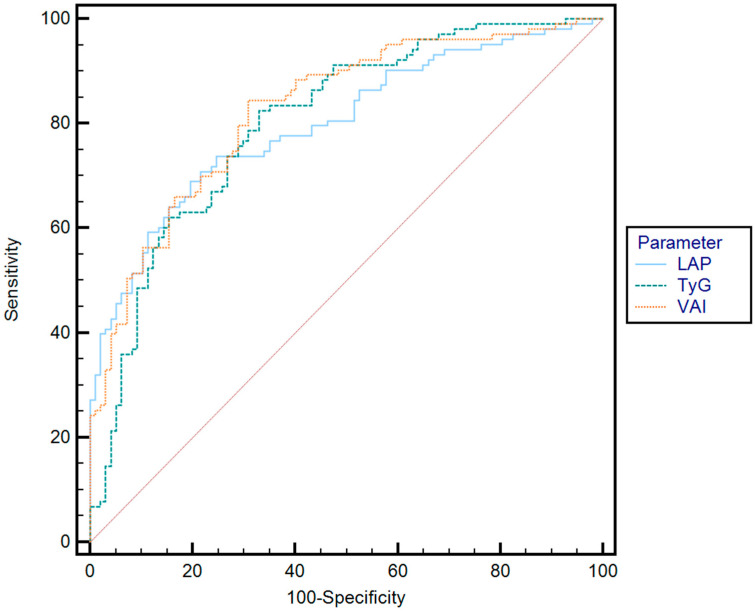
Comparison between receiver operating characteristics for LAP, TyG, and VAI for predicting the presence of S2–S3 steatosis.

The AUROCs of the biomarkers for predicting S2–S3 steatosis were as follows: TyG (AUROC = 0.80), VAI (AUROC = 0.83), and LAP (AUROC = 0.79). Although the VAI showed a slightly higher discriminative ability, the differences in predictive accuracy between the three biomarkers were not statistically significant: LAP vs. TyG (*p* = 0.9421), LAP vs. VAI (*p* = 0.5440), and TyG vs. VAI (*p* = 0.5672) (Figure 3).

**Figure 4:**
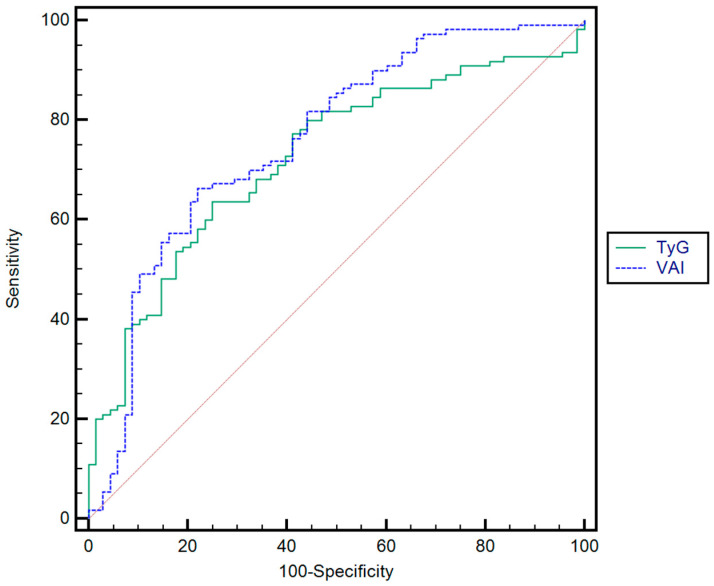
Comparison between receiver operating characteristics for TyG and VAI for predicting the presence of severe fibrosis.

The optimal cutoff values (largest sum of sensitivity [Se] and specificity [Sp]) for the TyG index and VAI for predicting the presence of severe fibrosis were as follows: 4.80 (AUROC = 0.72, Se = 82.8%, Sp = 62.3%, PPV = 61.5%, NPV = 84.1%) and 4.76 (AUROC = 0.76, Se = 81.3%, Sp = 61.3%, PPV = 63.8%, NPV = 82.2%), respectively. No statistically significant difference was observed between the diagnostic performance of the two parameters, with a *p*-value of 0.4854 (Figure 4). The rule-in and rule-out cut-off values for S3–S4 fibrosis are summarized in Table 7.

The NAFLD fibrosis score and FIB-4 score were calculated for all the included subjects. The rule-out and rule-in cut-off values for the NFS and FIB-4 were used for subject classification. To predict the absence of F3-F4 fibrosis, the rule-out cut-off values were used (NFS ≤ −1.455 and FIB-4 ≤ 1.3), and 51/114 (44.7%) of the subjects were classified as not having F3–F4 fibrosis, with 86.3% being correctly classified (classification by TE rule-in and rule-out cut-off values was used).

Using the rule-in cut-off values (NFS > 0.676 and FIB-4 > 2.67), 21/114 (18.4%) of the subjects were classified as having F3–F4 fibrosis, with 81% being correctly classified (classification by TE rule-in and rule-out cut-off values was used). However, 42/114 (36.8%) of the subjects had NFS and FIB-4 values between the proposed cut-off values (−1.455 ≤ NFS > 0.676 and 1.3 ≤ FIB-4 > 2.67) and remained unclassified. In these subjects, the TyG and VAI rule-in and rule-out cut-off values were used for classification, and 12/42 (28.5%) of them were additionally classified, with 75% (9/12) being correctly classified (Table 8). Using this approach the percentage of unclassified subjects decreased from 36.8% to 26.3%.

## 4. Discussion

Effectively managing the public health burden of obesity and related diseases, including MASLD, demands a collaborative approach involving both primary care providers and medical specialists across various fields, and it is vital to establish a simple and practical framework that leverages non-invasive testing methods, enabling the prompt and accurate identification of MASLD patients in need of specialized care.

The present study evaluated the diagnostic performance of three non-invasive markers (TyG, VAI, LAP) known to be associated with obesity-related and metabolic syndrome pathologies in identifying hepatic steatosis, aiming for the rapid and accurate detection of affected individuals. Furthermore, this study sought to identify patients with severe fibrosis—those at high risk of developing complications—to facilitate their referral to specialists and additional investigations.

Studies have primarily evaluated the performance of the TyG index for detecting complications of diabetes mellitus or cardiovascular-related conditions [17,23,24], while its role as a predictive marker for hepatic steatosis and fibrosis has been less extensively studied.

Existing evidence indicates that triglycerides and fasting blood glucose play a role in the development of fatty liver, with insulin resistance (IR) being a key factor in the pathogenesis of MAFLD. Furthermore, the TyG index has been strongly linked to NAFLD and is recognized as an effective, practical, and cost-efficient tool for identifying individuals at risk of hepatic steatosis, offering high sensitivity and specificity [25]. As such, the TyG index shows promise as a valuable diagnostic marker for MAFLD.

In our study, the TyG index values were significantly higher in subjects with severe steatosis, showing positive correlations with the degree of steatosis (r = 0.66) and fibrosis (r = 0.53). A recent study [21] revealed that the TyG and TyG-related indices were significantly associated with all-cause, cardiovascular, and diabetes-related mortality in adults with MASLD. Moreover, findings based on NHANES data demonstrated that the TyG index is strongly associated with hepatic steatosis and liver fibrosis even in non-diabetic, non-hypertensive individuals, showing superior predictive performance compared to conventional hepatic markers such as the FLI and FIB-4. These results highlight the potential of the TyG for early identification of liver disease risk in metabolically healthy populations [26]. Although in our results we did not provide data on the association between this parameter and mortality from various causes, the observed correlation between the TyG values and the degree of fibrosis suggests that it may predict more advanced liver disease. This, in turn, indicates a higher risk of liver-related complications and, in some cases, even death. Another recent study [27] that included 122 subjects with MASLD evaluated the performance of the TyG index for diagnosing hepatic steatosis, demonstrating an AUC of 0.77, similar to that found in our study: AUC of 0.80 for steatosis and 0.72 for fibrosis. These findings reinforce the results demonstrated by a systematic review and meta-analysis published in 2022, which showed that the TyG index has excellent performance for the diagnosis and prediction of hepatic steatosis, with an AUC of 0.75 [11].

Another parameter evaluated in this study is the visceral adiposity index, considering that previous studies have demonstrated the association between the VAI and the presence of hepatic steatosis [28,29,30,31,32]. The relationship between the VAI and NAFLD can be explained through a straightforward mechanism demonstrated in a recent study [33]. This study showed that larger amounts of visceral adipose tissue were consistently associated with an increased risk of developing NAFLD, proving that the fat distribution exerts a greater impact on NAFLD development than the fat content alone. The VAI acts as a simple and easily calculable indirect marker of visceral adipose tissue, including parameters that reflect and are closely linked to adipose tissue dysfunction and abnormal fat distribution. In a comprehensive and meticulously conducted meta-analysis that included 24 studies [34], the conclusions regarding the performance of the VAI in predicting hepatic steatosis were similar to those found in our study. Similar to our study, it was observed that the VAI values were significantly higher in patients with severe steatosis compared to those with simple steatosis. Regarding the performance of the VAI in predicting hepatic steatosis, our study demonstrated an AUC of 0.83, which is slightly higher than the value reported in the aforementioned meta-analysis. This meta-analysis included six studies that evaluated the VAI for predicting NAFLD and reported an overall AUC of 0.76.

The evaluation of the VAI in relation to the presence and prediction of fibrosis demonstrated a significant positive correlation with the presence of hepatic fibrosis (r = 0.63), as well as an association between the VAI values and the severity of hepatic fibrosis in multivariate analysis. This aligns with the conclusions of a study published by Petta et al. [35], which demonstrated that higher VAI values (OR 1.446, 95% CI 1.023–2.043, *p* = 0.03) were significantly associated with advanced fibrosis (F2–F4) in a multivariate logistic regression analysis. On the other hand, the results of studies are inconsistent, with some failing to demonstrate an association between the VAI and the presence or severity of hepatic fibrosis [36,37].

The lipid accumulation product (LAP) is another non-invasive marker regarded as an indirect indicator of lipid accumulation, which has drawn considerable research interest. A meta-analysis published in 2023 included 16 studies, of which 14 reported a significant association between the LAP index and NAFLD, while 2 found this relationship to be non-significant [35]. Although the studies were heterogeneous and their conclusions were not always consistent, the aforementioned meta-analysis concluded that the LAP index is an inexpensive, sensitive, and specific tool for evaluating NAFLD and may hold significant value for NAFLD screening. Similarly, in our study, a significant positive correlation was identified between the LAP values and the presence of hepatic steatosis (r = 0.61). In the multivariate analysis, an association with the presence of steatosis was observed but not with the presence of fibrosis. The performance for predicting steatosis proved to be good, with an AUC of 0.79, but it was significantly lower compared to that reported in Ebrahimi’s meta-analysis [38], where the summary AUC was 0.95.

There are limited data available regarding the performance of the LAP in predicting the presence of hepatic fibrosis. In fact, none of the aforementioned markers have consistent and validated studies supporting this aspect. Similar to our study, most studies have concluded that the VAI demonstrates the best performance in predicting hepatic fibrosis, with the evidence being strongly supported by previously published research.

In addition to the aspects discussed above, several recent studies have focused on more complex and innovative mechanisms for the prediction of hepatic steatosis and fibrosis. These studies not only reinforce the clinical value of existing indices but also open new perspectives for refining risk stratification and advancing early disease detection.

A recent study showed that ubiquitin-specific protease 29 (USP29) acts as a protective factor against metabolic dysfunction-associated steatotic liver disease (MASLD) progression by stabilizing long-chain acyl-CoA synthase 5 (ACSL5) and enhancing fatty acid β-oxidation, suggesting that the activation of the USP29–ACSL5 axis could represent a promising therapeutic strategy for MASLD [39].

Another promising research direction involves the role of circulating microRNAs (miRNAs) as non-invasive diagnostic tools. A comprehensive review [40] summarized the current evidence and showed that specific miRNA profiles are closely associated with various digestive diseases, including MASLD. miRNAs regulate key pathways involved in inflammation, lipid metabolism, and fibrogenesis. Thus, miRNA panels could, in the near future, complement existing non-invasive indices such as the TyG, VAI, and LAP, improving diagnostic precision and disease monitoring. Although not evaluated in our current study, miRNAs represent an emerging and exciting field that deserves future exploration, particularly in the context of MASLD and hepatic fibrosis.

Ultimately, as artificial intelligence technologies have become increasingly integrated into all aspects of healthcare, recent studies have also explored their application in the diagnosis, risk stratification, and management of metabolic dysfunction-associated steatotic liver disease (MASLD), offering new opportunities for earlier detection and personalized treatment strategies [41].

While these innovative approaches represent promising future directions for enhancing diagnostic precision and personalized care, simpler and easily accessible clinical markers remain practical tools for rapid patient stratification in daily practice, particularly for assessing the risk of significant steatosis and fibrosis.

Building on these considerations, our study further demonstrates how combining simple clinical markers can enhance the accuracy of steatosis and fibrosis assessment. An important finding of the present study is that integrating the TyG and VAI rule-in and rule-out cut-off values as complementary tools to the NFS and FIB-4 significantly improved the classification accuracy, reducing the proportion of unclassified subjects from 36.8% to 26.3%. This underscores the utility of these indices in refining fibrosis assessment and enabling more precise patient stratification, particularly in cases where conventional scores yield inconclusive results.

However, although studies show agreement regarding the performance of these markers in predicting hepatic steatosis, the findings related to fibrosis are inconsistent. This highlights the need for further evaluation of these markers in terms of their predictive performance for hepatic fibrosis. Future studies should include larger cohorts and ideally use liver biopsy as the reference standard for fibrosis staging.

## 5. Conclusions

In conclusion, our study demonstrates that incorporating the TyG and VAI cut-off values alongside conventional scores such as the NFS and FIB-4 improves the classification accuracy of hepatic steatosis and fibrosis. This combined approach reduced the proportion of unclassified patients from 36.8% to 26.3%, highlighting its potential utility in clinical practice. Although the individual performance of the TyG and VAI in fibrosis detection remains to be fully validated, their integration into existing scoring strategies enhances diagnostic precision and supports better patient stratification. These findings suggest that using the TyG and VAI may help identify patients at higher risk of advanced liver disease who warrant further evaluation and specialist referral.

## Figures and Tables

**Figure 2:**
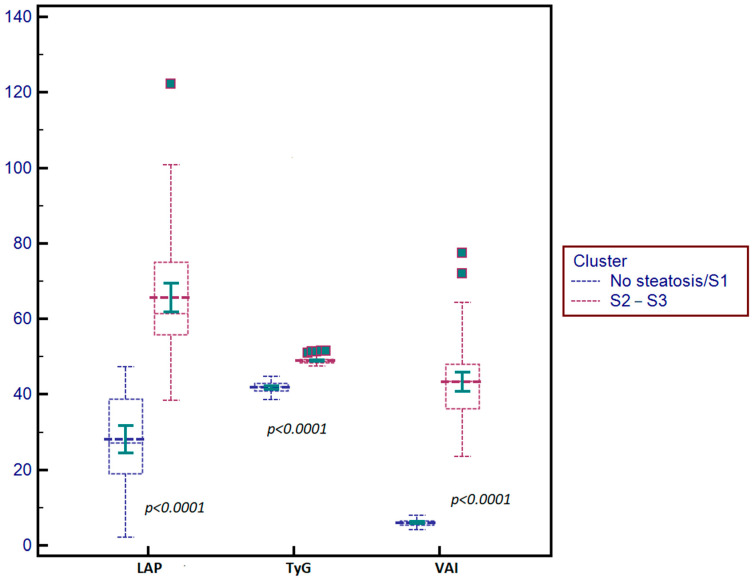
Box-and-whisker distribution plots comparing the LAP, TyG, and VAI values according to the presence and grade of liver steatosis.

**Table 1:** Laboratory parameters evaluated from fasting venous blood samples.

Parameter	Methodology
Complete Blood Count (CBC)	Automated hematology analyzer
Fasting Blood Glucose	Enzymatic hexokinase method
Total Cholesterol	Enzymatic colorimetric method
HDL-Cholesterol	Direct enzymatic method
LDL-Cholesterol	Calculated using the Friedewald formula (if TG < 400 mg/dL) or direct measurement
Triglycerides	Enzymatic colorimetric method
Gamma-Glutamyl Transferase (γ-GT)	Enzymatic method
Serum Albumin	Bromocresol green dye-binding method
AST, ALT	IFCC (International Federation of Clinical Chemistry) standardized enzymatic methods

**Table 2:** Patients’ characteristics.

Parameter	n = 178
Age	52.79 ± 12.56
Gender	
Males	98 (55.1%)
Females	80 (44.9%)
BMI (kg/m^2^)	31.4 ± 5.3
Abdominal circumference (AC) (cm)	109.1 ± 11.4
Laboratory findings	
RBCs (g/dL)	11.1 ± 3.76
WBCs (×10⁹/L)	8.21 ± 2.49
Platelet count (×10⁹/L)	225 ± 38.7
Serum albumin levels (mg/dL)	3.3 ± 1.23
AST (UI/L)	42.3 ± 23.7
ALT (UI/L)	53.1 ± 39.6
Cholesterol (mg/dL)	203.2 ± 46.6
HDLc (mg/dL)	44.3 ± 10.6
LDLc (mg/dL)	149 ± 32.8
Triglyceride (mg/dL)	198.2 ± 148.9
FBG (mg/dL)	114.5 [110–120]
Liver steatosis distribution by CAP	
CAP mean values (dB/m)	322.5 ± 39.6
S0	55 (31%)
S1	9 (5%)
S2	38 (21.3%)
S3	76 (42.7%)
Liver fibrosis distribution by TE	
F0–F1	81 (45.5%)
F2	48 (27%)
F3	31 (17.4%)
F4	18 (10.1%)
Visceral adiposity non-invasive biomarkers	
TyG index	4.93 [4.82–5.31]
VAI	3.67 [2.51–4.63]
LAP	74.02 [61.34–86.29]

Numerical variables with normal distribution are presented as means ± standard deviations, while variables with non-normal distribution are presented as median values and ranges. BMI—body mass index, n—number, AC—Abdominal circumference, RBCs—red blood cells, WBCs—white blood cells, AST—Aspartate Aminotransferase, ALT—Alanine Aminotransferase, FBG—fasting blood glucose, HDLc—high-density lipoprotein cholesterol, LDLc—low-density lipoprotein cholesterol, LAP—lipid accumulation product, VAI—visceral adiposity index, TyG index—triglyceride–glucose Index, S0—without steatosis, S1—mild steatosis, S2—moderate steatosis, S3—severe steatosis, CAP—controlled attenuation parameter.

**Table 3:** Comparison of steatosis and fibrosis groups according to age and gender.

Parameter	S0–S1 (n = 64)	S2–S3 (n = 114)	*p* Value	F0–F2 (n = 129)	F3–F4 (n = 49)	*p* Value
Gender distribution						
Male	57.8% (37)	53.5% (61)	0.6981	53.5% (69)	59.2% (29)	0.6071
Female	42.2% (27)	46.5% (53)	0.6981	46.5% (60)	40.8% (20)	0.6071
Mean age	53.49 ± 9.98	52.44 ± 12.2	0.5581	54.22 ± 10.11	51.3 ± 11.6	0.1001

The TyG index, VAI, and LAP mean values were significantly higher in subjects with S2–S3 steatosis compared to those without steatosis or S1 steatosis, *p* < 0.0001 (Table 4). The distributions of the TyG, VAI, and LAP values according to the grade of steatosis are detailed in Figure 2.

**Table 4:** Differences between TyG, VAI, and LAP values in subjects with S2–S3 steatosis compared to those without.

Parameter	S0–S1	S2–S3	*p* Value
TyG index	4.20 ± 0.32	4.87 ± 0.43	*p* < 0.0001
VAI	1.09 ± 0.22	4.35 ± 0.56	*p* < 0.0001
LAP	25.40 ± 6.32	61.41 ±12.13	*p* < 0.0001

LAP—lipid accumulation product, VAI—visceral adiposity index, TyG index—triglyceride–glucose index.

**Table 5:** Associations between TyG index, VAI, and LAP and liver steatosis/fibrosis.

Parameter	Liver Steatosis	Liver Fibrosis
TyG index	r = 0.66, *p* < 0.0001 95% CI [0.491–0.742]	r = 0.53, *p* < 0.0001 95% CI [0.411–0.623]
VAI	r = 0.76, *p* < 0.0001 95% CI [0.641–0.822]	r = 0.63, *p* < 0.0001 95% CI [0.511–0.702]
LAP	r = 0.61, *p* < 0.0001 95% CI [0.481–0.683]	r = 0.51, *p* < 0.0001 95% CI [0.381–0.542]

LAP—lipid accumulation product, VAI—visceral adiposity index, TyG index—triglyceride–glucose index, 95% CI—95% confidence interval.

**Table 6:** Multivariate regression analysis models of liver steatosis and liver fibrosis.

Predictor	Regression Parameters				
	β	SE	*p*	OR	95% CI
Multivariate regression analysis of liver steatosis					
AC (cm)	β = 1.006	±0.083	*p* = 0.0381	OR = 2.734	[2.331–3.207]
BMI (kg/m^2^)	β = 1.062	±0.091	*p* = 0.0414	OR = 2.892	[2.457–3.402]
TyG	β = 2.136	±0.58	*p* = 0.0033	OR = 8.467	[2.730–26.3]
VAI	β = 2.044	±0.91	*p* < 0.0001	OR = 7.721	[1.297–45.95]
LAP	β = 1.022	±0.095	*p* = 0.0341	OR = 2.778	[2.303–3.351]
Multivariate regression analysis of liver fibrosis					
TyG	β = 1.936	±0.88	*p* = 0.0112	OR = 6.927	[1.226–39.15]
VAI	β = 2.009	±0.91	*p* = 0.002	OR = 7.456	[1.260–44.12]

LAP—lipid accumulation product, VAI—visceral adiposity index, TyG index—triglyceride–glucose index, SE—standard error, 95% CI—95% confidence interval, OR—odds ratio, β—beta coefficient.

**Table 7:** Optimal, rule-in, and rule-out cut-off values for predicting severe fibrosis (F3–F4).

	Parameter	Cut-Off	AUC	Se (%)	Sp (%)	PPV (%)	NPV (%)	*p*
Optimal cut-off value *	TyG	4.80	0.72	82.8	62.3	61.5	84.1	*p* < 0.001
	VAI	4.76	0.76	81.3	61.3	63.8	82.2	*p* < 0.001
Rule-in **	TyG	5.61	0.72	49	74	72.9	63	*p* < 0.001
	VAI	6.21	0.76	53.9	79	2.6	72.6	*p* < 0.001
Rule-out ***	TyG	2.65	0.72	80.3	41.6	52.7	83.6	*p* < 0.001
	VAI	1.67	0.76	81.2	50	56.5	87.7	*p* < 0.001

VAI—visceral adiposity index, TyG index—triglyceride–glucose index, Se—sensitivity; Sp—specificity; PPV—positive predictive value; NPV—negative predictive value; AUC—area under the curve. * Cut-off values with the higher sum of sensitivity and specificity were chosen. ** Cut-off values that optimized specificity were chosen. *** Cut-off values that optimized sensitivity were chosen.

**Table 8:** Classification of subjects based on rule-out and rule-in cutoff values.

		Grey zone	
	Rule out F3–F4 NFS ≤ −1.455 and FIB-4 ≤ 1.3	−1.455 ≤ NFS > 0.676 1.3 ≤ FIB-4 > 2.67	Rule in F3–F4 NFS > 0.676 and FIB-4 > 2.67
	Absence of F3–F4	Unclassified subjects	Presence of F3–F4
Classification by NFS and FIB-4 rule-in and rule-out cut-off values	51/114 (44.7%)	42/114 (36.8%)	21/114 (18.4%)
Classification by TE rule-in and rule-out cut-off values	44/51 (86.3%) correctly classified		17/21(81%) correctly classified
Classification by TyG and VAI rule-in and rule-out cut-off values		12/42 (28.5%) additionally classified, with 75% (9/12) of them correctly classified	

## Data Availability

Data are available upon request.
